# Continuous Process Verification 4.0 application in upstream: adaptiveness implementation managed by AI in the hypoxic bioprocess of the *Pichia pastoris* cell factory

**DOI:** 10.3389/fbioe.2024.1439638

**Published:** 2024-10-02

**Authors:** Arnau Gasset, Joeri Van Wijngaarden, Ferran Mirabent, Albert Sales-Vallverdú, Xavier Garcia-Ortega, José Luis Montesinos-Seguí, Toni Manzano, Francisco Valero

**Affiliations:** ^1^ Department of Chemical, Biological, and Environmental Engineering, School of Engineering, Universitat Autònoma de Barcelona, Bellaterra, Barcelona, Spain; ^2^ Aizon, Barcelona, Spain

**Keywords:** artificial intelligence, industry 4.0, continued process verification, digital twin, physiological control, respiratory quotient, *Pichia pastoris*, recombinant protein production

## Abstract

The experimental approach developed in this research demonstrated how the cloud, the Internet of Things (IoT), edge computing, and Artificial Intelligence (AI), considered key technologies in Industry 4.0, provide the expected horizon for adaptive vision in Continued Process Verification (CPV), the final stage of Process Validation (PV). *Pichia pastoris* producing *Candida rugosa* lipase 1 under the regulation of the constitutive *GAP* promoter was selected as an experimental bioprocess. The bioprocess worked under hypoxic conditions in carbon-limited fed-batch cultures through a physiological control based on the respiratory quotient (*RQ*). In this novel bioprocess, a digital twin (DT) was built and successfully tested. The implementation of online sensors worked as a bridge between the microorganism and AI models, to provide predictions from the edge and the cloud. AI models emulated the metabolism of *Pichia* based on critical process parameters and actionable factors to achieve the expected quality attributes. This innovative AI-aided Adaptive-Proportional Control strategy (AI-APC) improved the reproducibility comparing to a Manual-Heuristic Control strategy (MHC), showing better performance than the Boolean-Logic-Controller (BLC) tested. The accuracy, indicated by the Mean Relative Error (MRE), was for the AI-APC lower than 4%, better than the obtained for MHC (10%) and BLC (5%). Moreover, in terms of precision, the same trend was observed when comparing the Root Mean Square Deviation (RMSD) values, becoming lower as the complexity of the controller increases. The successful automatic real time control of the bioprocess orchestrated by AI models proved the 4.0 capabilities brought by the adaptive concept and its validity in biopharmaceutical upstream operations.

## 1 Introduction

**FIGURE 1:**
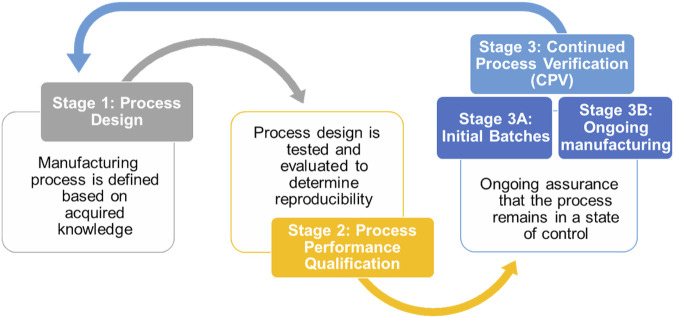
Process Validation stages proposed by the FDA (FDA, 2011) as good practices for drug development and manufacturing.

In the pharmaceutical manufacturing context, the US Food and Drug Administration (FDA) defines process validation (PV) as the systematic collection and evaluation of data throughout the entire product lifecycle, from process design to commercial production, demonstrating the consistent production of a quality product (FDA, 2011). This process involves three sequential stages: Stage 1 focuses on Process Design (PD), defining the commercial manufacturing process, including key conditions for quality. Stage 2, or Process Qualification (PQ), evaluates the PD for capability and reproducibility. Introduced in 2011, Stage 3, Continued Process Verification (CPV), ensures ongoing quality assurance during routine production through real-time data monitoring. Concurrently, the concept of Industry 4.0 emerged in 2011, aiming to enhance manufacturing competitiveness through cyber-systems (Manzano and Whitford, 2023; Steinwandter et al., 2019). The European Medicines Agency (EMA) emphasises the importance of PV guidelines for pharmaceutical manufacturers from Stage 1 to batch release (EMA, 2014). CPV ensures control through real-time data and direct interaction with production elements. Stage 2 verifies drug manufacturing processes; however, uncertainties arise in the production environment due to equipment stress, seasonality, shifts, and raw material variations. Established Conditions (EC), Critical Material Attributes (CMA), Critical Process Parameters (CPP), and Critical Quality Attributes (CQA) from Stages 1 and 2 may not capture all aspects of the manufacturing process. Stage 3 of PV is divided into an initial step (Stage 3A) for preliminary production assessment, and a subsequent phase (Stage 3B) based on experienced iterations with robust conditions after successful batch releases (Figure 1).

Digital Twins (DT) can be defined as virtual replicas of physical systems, utilising real-time data processed in cloud systems to emulate and mirror the behaviour of the actual objects (Bao et al., 2019). In biopharmaceuticals, the emulation of biophysical systems through Artificial Intelligence (AI) is a known technique. AI, employing a multivariable approach, offers statistical capabilities to detect unexpected interactions and predict complex interconnected factors, affording control over intricate processes with numerous variables. AI’s proficiency in tasks like image recognition, multivariate prediction, and fast classifications is particularly advantageous in drug manufacturing, where human interactions can be the root cause of issues (Cintron, 2015). AI is effectively applied to tasks such as anomaly detection in continuous automated processes and the identification of defects (Ündey et al., 2010). However, challenges exist in the continuous adaptation of equipment and tasks based on real-time data in the biopharmaceutical setting (Ündey et al., 2010).

Regulatory bodies advocate for the use of multivariable statistical approaches, particularly at Stage 3 of CPV, to ensure correct batch production and reproducibility by implementing non-invasive sensors during production, known as Process Analytical Technologies (PAT) (Read et al., 2010). In fact, both the EMA and FDA specify that reproducibility is an essential aspect of the PV of the manufacture of biotechnological or biomedical products (EMA, 2014; FDA, 2011). Despite these recommendations, broad deployment of PAT is still lacking. The European Pharmacopoeia recognises AI algorithms like Neural Networks and Support Vector Machines as valid chemometric methods for pharmaceutical contexts (Council of Europe, 2017). The FDA has also outlined a methodology for AI application in medical devices (FDA, 2019).

Besides offering a way of achieving improved reproducibility, automation is considered a crucial step in implementing complex industrial bioprocesses, enhancing product quality, reliability, and economic efficiency (Manzano et al., 2021). It is particularly essential in dealing with process failures appropriately (Alford, 2006; Rathore et al., 2021; Stanke and Hitzmann, 2013). Aside from improving product quality and safety, automation also enhances human safety by reducing the number of manual control activities required by plant operators, which can be potentially dangerous or jeopardise the entire bioprocess (Clementschitsch and Bayer, 2006; Luo et al., 2021). Despite encouragement from regulatory bodies (EMA, 2014; Embury and Clayton, 2017; FDA, 2011), the biotech and biopharma industries are reluctant to embrace automation with novel control strategies, including AI, due to associated re-validation steps (Sahiner et al., 2018). Noteworthy, reviews on the role of big data in industrial (Bio)chemical process operations have been published recently (ICH, 2009; Udugama et al., 2020).

In industrial biotechnology, the yeast *Komagataella phaffii* (formerly known as *Pichia pastoris*) is currently one of the most used microbial cell factories for the production of both chemicals and recombinant proteins for a wide range of applications, from biomaterials to biopharmaceuticals (García-Ortega et al., 2019). Accordingly, a bioprocess based on this cell factory for recombinant protein production (RPP) was selected as a model to implement the concepts of CPV and Industry 4.0 due to relevant advantages over other yeast cell factories (Ata et al., 2021; Duman-Özdamar and Binay, 2021; Eskandari et al., 2024; Khlebodarova et al., 2024).

Previous studies have shown a clear increase in RPP, specifically in continuous and fed-batch bioprocesses producing an antibody fragment (Fab) and the enzyme *Candida rugosa* Lipase 1 (Crl1) under *GAP* promoter (*P*_*GAP*_) regulation in hypoxic conditions when glucose was used as the carbon source. This production increase could be explained by transcriptional analyses in which glycolytic genes expression was significantly upregulated (Baumann et al., 2010; Garcia-Ortega et al., 2017; Gasset et al., 2022; Sales-Vallverdú et al., 2024).

From an industrial perspective, reproducibility is crucial when implementing a productive bioprocess (Galvanauskas et al., 2019; Simutis and Lübbert, 2015; Veloso and Ferreira, 2017). Working under hypoxic controlled conditions in continuous and/or fed-batch bioprocesses is not a standard classic strategy and, ideally, the methodology should be transferrable between fermentation systems with different oxygen-transfer capacities and be scalable. Thus, accurate monitoring and control of the respiratory quotient (*RQ*) is needed to maintain a constant and reproducible hypoxic state. Furthermore, selecting a suitable *RQ* set-point is equally essential to ensure the desired results.

As previous works have shown (Gasset et al., 2022; Sales-Vallverdú et al., 2024), the selection of the appropriate *RQ* set-point can be summarised in the following two points: first, hypoxic conditions can be assured if *RQ* > 1.2, so a higher set-point should be selected. Secondly, the higher the *RQ*, the greater the ethanol production and the lower the biomass-to-substrate yield (*Y*_*X/S*_), both factors affecting the efficiency of the bioprocess. Specifically, the production of excessive amounts of ethanol can result in growth and RPP inhibition (Ergün et al., 2019; Wehbe et al., 2020). Taking into account these considerations, *RQ* should be maintained within the range of 1.2–1.6, with 1.4 being a suitable value to be defined as the set-point.

Pattern recognition and outlier detection mechanisms have also been implemented to detect potential failures or unexpected behaviour during the process. AI modelling based on supervised random forest models was used for the prediction of the required operator’s control actions to maintain the optimal set-point of the *RQ* (Ondracka et al., 2023).

These previous works were suitable for conducting an exploratory study to quantify the production increase working under hypoxic versus normoxic conditions, and the effectiveness of the application of DTs in upstream biomanufacturing operations for bioindustries. However, further approaches will be required to implement the concept of CPV in this bioprocess.

Thus, the objectives of this work were, in a first attempt, to develop a manual-heuristic control (MHC) of *RQ*. This MHC was subsequently improved by applying a Boolean-logic control strategy (BLC). However, the main objective was to demonstrate the value of AI in guiding an upstream process based on the RPP of the *Pichia pastoris* cell factory. The autonomous control of the bioprocess in real time orchestrated by several AI models and its comparison with MHC and BLC strategies was evaluated. Additionally, a DT was employed as a bridge between the microorganism and AI models, to provide predictions from the edge and also from the cloud.

The experimental approach developed in this research aimed to demonstrate how the cloud, the Internet of Things (IoT), edge computing, and AI, considered key technologies in Industry 4.0, can be successfully applied to improve bioprocesses based on innovative control strategies. In this sense, by using AI to continuously monitor and control in real-time, CPV would help to maintain optimal conditions, ensuring robustness and reliability in the hypoxic bioprocess of *Pichia pastoris*.

## 2 Materials and methods

### 2.1 Recombinant expressing strain

One single-copy producer clone of *P. pastoris* derived from strain X-33 recombinantly expressing the *C. rugosa* lipase 1 (Crl1) under the regulation of the *GAP* promoter (*P*_*GAP*_) was used in this study. Details about strain constructions and clonal screening were published previously (Nieto-Taype et al., 2020).

### 2.2 Fed-batch cultivations

Two independent replicates of fed-batch cultivations with each of the three control strategies, Manual-Heuristic Control (MHC), Boolean-Logic Control (BLC), and AI-aided Adaptive Proportional Control (AI-APC), were performed using a 5 L Biostat B fermenter (Sartorius Stedim, Göttingen, Germany). The inoculum preparation for these fermentations is described in detail elsewhere (Sales-Vallverdú et al., 2024).

The fermentations were initiated with a batch phase, using glycerol as the carbon source. Batch media composition is described in the literature (Garcia-Ortega et al., 2016). Throughout the cultivations, temperature and pH were kept constant at 25°C and 6.0, respectively. In the fed-batch phase, the specific growth rate (*μ*) was fixed at 0.10 h^-1^ by applying an exponential pre-programmed glucose feeding profile, thus maintaining C-limiting conditions. The general operating procedure and the fed-batch media composition are further detailed in the literature (Garcia-Ortega et al., 2016; Gasset et al., 2022).

Since the study aimed to compare the performance of different control strategies for maintaining equivalent hypoxic conditions, the duration of all fed-batch phases was quite similar, and the biomass production was comparable. The desired level of oxygen limitation was achieved by maintaining the *RQ* at a set-point value of around 1.4. To do so, the agitation rate was modified to increase or reduce the oxygen transfer rate (*OTR*) and thus, the oxygen uptake rate (*OUR*). This modification was implemented following different strategies further detailed in the results section.

In this work, *Eve* software from INFORS HT (Bottmingen, Switzerland) was used as a “Supervisory Control And Data Acquisition” or “SCADA” system to integrate all data from the bioreactor and peripheral analysers. It allowed the implementation of the *RQ* soft sensor used in the BLC and AI-APC strategies.

### 2.3 Biomass analysis

Biomass concentration was determined as dry cell weight (DCW) in four independent replicates with the methodology described previously (Cos et al., 2005). The relative standard deviation (RSD) was always below 5%. Additionally, the elemental composition of biomass was also analysed using an established protocol (Cos et al., 2005).

### 2.4 Gas analysis

Using a BlueInOne FERM gas analyser (BlueSens, Herten, Germany), CO_2_ and O_2_ molar fractions, as well as humidity, were monitored online from the fermenter off-gas, and intermittently measured from inlet gas. To ensure accurate measurements, a recalibration of the gas analyser was conducted at each fermentation. Furthermore, humidity was minimised using a silica column before the analyser to enhance measurement precision. The obtained data enabled the calculation of respirometric parameters: oxygen uptake rate (*OUR)*, carbon dioxide evolution rate (*CER*), and respiratory quotient (*RQ)*, along with their corresponding specific rates (*q*_*O2*_ and *q*_*CO2*_). The RSD was estimated below 5%.

### 2.5 Substrate and by-product analysis

The carbon sources of the batch and fed-batch phases (glycerol and glucose, respectively) and the fermentation by-products (ethanol, arabitol, and succinate) were quantified using HPLC. The column and software were described previously (Jordà et al., 2014). The RSD of this analysis was below 2%.

### 2.6 Product analysis

Crl1 lipolytic activity was determined using the p-nitrophenyl butyrate (pNPB) assay described in detail in the literature (Garrigós-Martínez et al., 2019). One activity unit (AU) is defined as the amount of enzyme necessary to generate 1 µmol of product per minute under assay conditions. Crl1 titres in this work are given in kilo-AU per litre (kAU·L^-1^). The RSD was below 1%.

### 2.7 Process parameters and data consistency check

Through a combination of online and offline measurements, seven key specific rates were determined in the black-box process model. Notably, lipase production was considered negligible for the carbon and redox balances. The specific rates determined included growth (*μ*), substrate consumption (*q*_*S*_), oxygen uptake (*q*_*O2*_), carbon dioxide production (*q*_*CO2*_), as well as ethanol, arabitol, and succinate production as fermentation by-products (*q*_*EtOH*_, *q*_*Ara*_, and *q*_*Suc*_, respectively).

In all fed-batch cultivations, the data indicated that the closing of the carbon balance generally deviated less than 5%. To ensure consistency in the measurements, the experimental data was then verified using previously described data consistency and reconciliation protocols, applying the constraint that carbon and electron balances must be fulfilled (Ponte et al., 2016; Wang and Stephanopoulos, 1983). A confidence level of 95% was reached in all statistical consistency tests, indicating that no significant measurement errors were made.

## 3 Results and discussion

### 3.1 Selection of the manipulated variable for hypoxic control

Carbon-limited fed-batch fermentations exhibit an exponential increase in total biomass, product titre, and all other growth-related components, and they are defined by an exponential increase in oxygen consumption and carbon dioxide production. Then, any control action performed over this process must be time-dependent with a nonlinear behaviour.

A variable with a high impact on *RQ* should be used for control strategy implementation. Some examples can be found in the literature where respirometric parameters were used as measured variables to implement advanced control strategies. Nonetheless, in these studies, the modified variable was feeding addition, therefore, not maintaining a constant *μ* value (Mesquita et al., 2019; Ranjan and Gomes, 2009; Wang et al., 2007). If the objective also includes keeping *μ* constant, then another manipulated variable or variables should be selected for the *RQ* control strategy.

As shown in Equations 1–3, biological and operational variables affecting *RQ* can be stated as *q*_*O2*_, *q*_*CO2*_, and *X*.

$$CER=q_{CO2}\cdot X$$ (1)

$$OUR=q_{O2}\cdot X$$ (2)

$$RQ=\frac{CER}{OUR}$$ (3)

Where *CER* and *OUR* are the Carbon Evolution Rate (mol CO_2_·L^-1^·h^-1^) and Oxygen Uptake Rate (mol O_2_·L^-1^·h^-1^), respectively; *q*_*CO2*_ and *q*_*O2*_ are the specific carbon dioxide production rate (mol CO_2_·g_DCW_^-1^·h^-1^) and the specific oxygen consumption rate (mol O_2_·g_DCW_^-1^·h^-1^), respectively; *X* is biomass concentration (g_DCW_·L^-1^); and finally, *RQ* stands for Respiratory Quotient (mol CO_2_·mol O_2_^−1^, normally expressed as a non-dimensional variable).

In an oxygen-limited system, all the oxygen transferred to the culture is ideally supposed to be consumed immediately by the cells, so the *OTR* is considered equal to *OUR* (mol O_2_·L^-1^·h^-1^) (Equation 4).

OTR=OUR (4)

Then, it may be possible to modify the *RQ* by altering the oxygen availability described by *OTR*, although this can also have an impact on *CER*.

Equation 5 shows the variables involved in the *OTR* term:

$$OTR=k_{L}a\cdot \left({O_{2}}^{Sat}-O_{2}\right)$$ (5)

where *k*_*L*_ *a* is the volumetric mass transfer coefficient (h^-1^, or more commonly s^-1^), *O*_*2*_^*Sat*^ is the saturation oxygen concentration in the culture broth (mol O_2_·L^-1^), and *O*_*2*_ is the real oxygen concentration (mol O_2_·L^-1^). Under hypoxic conditions, *O*_*2*_ ≈ 0, so two parameters can be considered for *OTR* adjustment: *k*_*L*_ *a* and *O*_*2*_^*Sat*^.

Therefore, two different variables can be mainly proposed as manipulated variables to implement *RQ* control: agitation rate and oxygen molar fraction in the inlet gas. The former has a significant impact on *k*_*L*_ *a* since it affects both the turbulent regime in the bioreactor (*k*_*L*_) and the interfacial area between the gas and liquid phases (*a*); whereas the latter influences oxygen saturation in the culture broth (*O*_*2*_^*Sat*^) (Garcia-Ochoa et al., 2010). Nevertheless, from an industrial perspective, the use of pure gases is not very attractive as it entails additional transport and storage costs, safety risks, etc. (Liu et al., 2016). For this reason, the agitation rate was initially selected as the manipulated variable to implement *RQ* control.

### 3.2 A first approach to *RQ* control: manual-heuristic modification of the agitation rate (MHC)

**FIGURE 2:**
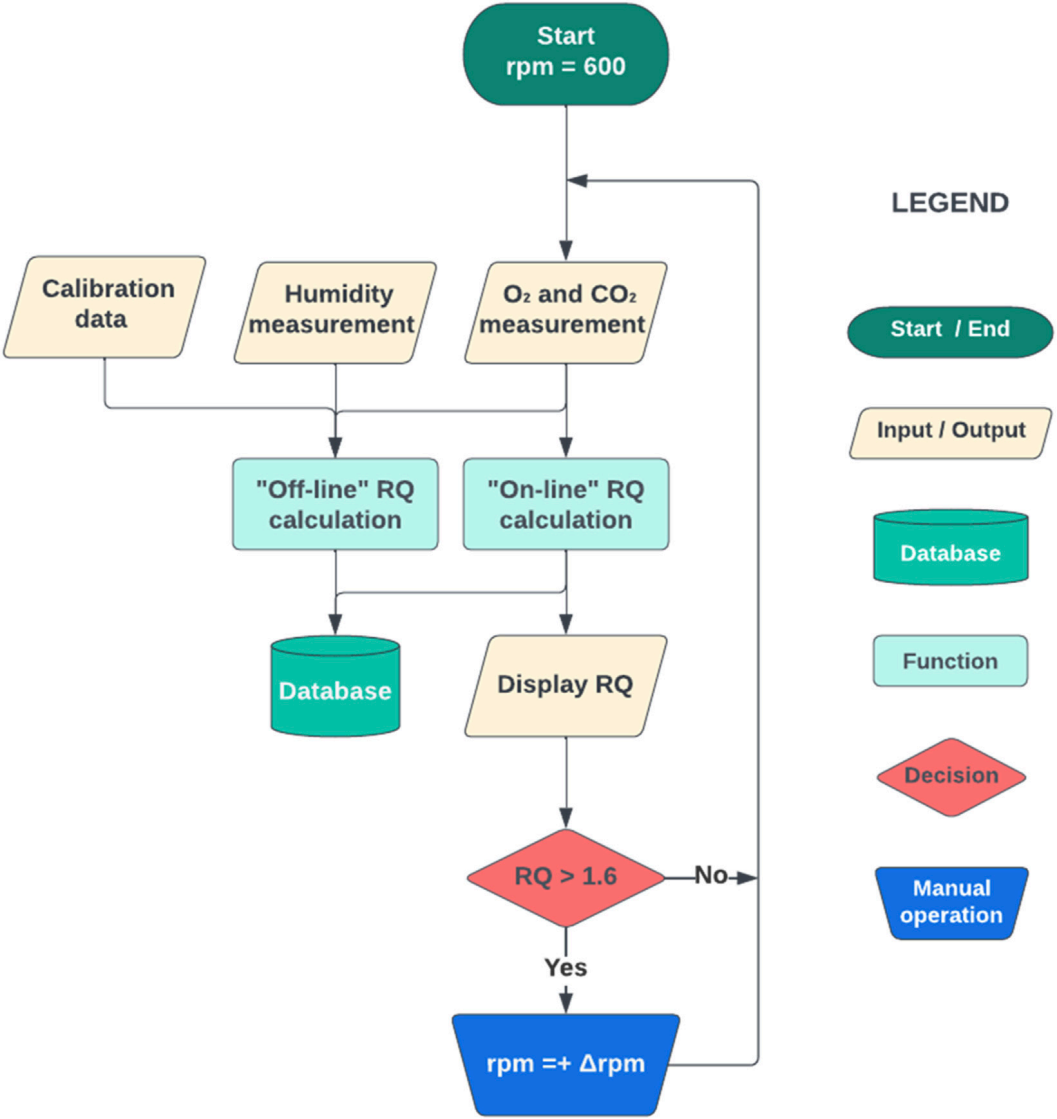
Flowchart of the manual-heuristic control strategy (MHC). The initial agitation rate was set at 600 rpm. *RQ* was calculated (online *RQ* calculation) using O_2_ and CO_2_ measurements, and every 15/30/60 min (variable time) the agitation rate was increased by 25/50/100 rpm (variable Δrpm), only if *RQ* > 1.6. This control strategy was manually implemented step by step, using *BlueVis* software (BlueSens, Herten, Germany) to calculate the *RQ* and the *BiostatB* interface (Sartorius, Göttingen, Germany) to increase the agitation rate.

The first approach to *RQ* control was to perform manual control actions on the agitation rate. Thus, two hypoxic fermentations were carried out as biological replicates to assess the viability of using the agitation rate as a modified variable to achieve precise and accurate *RQ* control. The manual modification of the agitation rate followed heuristic rules: increasing the agitation rate caused a reduction in *RQ*, thus control actions were based on this principle. An operational *RQ* range was defined as 1.2 < *RQ* < 1.6, and an initial agitation rate was set by trial and error to achieve an *RQ =* 1.4, being 600 rpm at the start of the fed-batch. Then, approximately every 30 min after initiating the fed-batch, the *RQ* was checked. If *RQ* > 1.6, then the agitation rate was increased by a step (∆rpm) of either 50 or 100 rpm, depending on the researcher’s expertise. Since the dynamics of the system was that the substrate uptake rate (*SUR*) grew exponentially as substrate addition increased and correspondingly total biomass, *CER* also increased exponentially. To maintain a constant *RQ*, *OTR* should also increase exponentially since *OUR* is coupled with biomass growth. Accordingly, the agitation rate should follow a similar nonlinear increasing trend. Reductions in the agitation rate during the fed-batch phase were therefore not expected. A flowchart of the MHC algorithm is shown in Figure 2.

The results of these hypoxic fermentations have been partially reported together with the results of two replicates of fully aerobic fermentations (DO>30%) (Gasset et al., 2022). This set of fermentations was performed to evaluate the effect of hypoxic conditions on RPP, which was fully demonstrated. However, apart from these two aforementioned objectives, these experiments also had a less apparent, yet equally relevant goal, which was to generate sufficient data to subsequently build an *RQ* control model using AI algorithms. This requires as much data as possible, to be trained with the aim of achieving accurate and feasible models (Das et al., 2015; Ondracka et al., 2023; Zhou et al., 2019). This question will be further discussed in the following sections.

**FIGURE 3:**
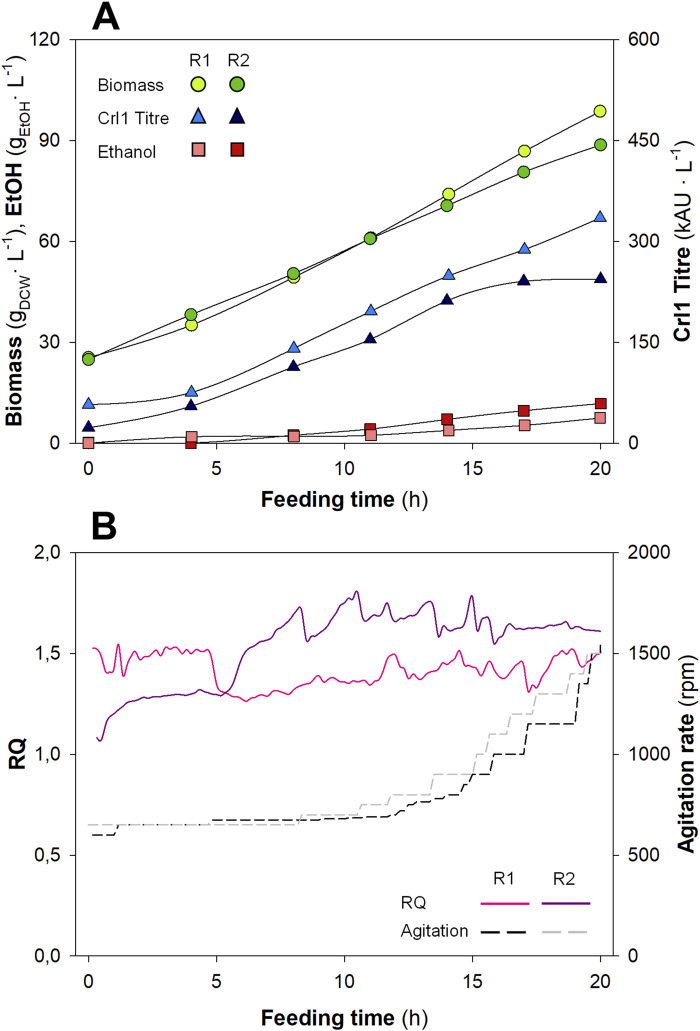
Key process parameters (Biomass, Ethanol, and Crl1), agitation rate, and *RQ* for the biological replicates (R1 and R2) with the Manual Heuristic control strategy (MHC). **(A)** Biomass concentration (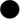, g_DCW_·L^-1^); Crl1 Titre (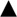, kAU·L^-1^); EtOH, Ethanol concentration (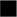, g·L^-1^). **(B)** Off-line *RQ* calculation (continuous lines); agitation rate (discontinuous lines, rpm).

Biomass and ethanol concentrations, as well as Crl1 titres, are plotted in Figure 3A, whereas RQ and agitation rate profiles are plotted in Figure 3B. Each replicate is plotted separately to evaluate the reproducibility between duplicates, which appears to be low. Biomass concentrations increased from ≈ 25 g_DCW_·L^-1^ (value at the end of the batch phase) to ≈ 90 g_DCW_·L^-1^ (R1) and ≈ 100 g_DCW_·L^-1^ (R2), showing some variability between replicates. Ethanol was not detectable at the beginning of the fed-batch phase; at the end of the fermentation, it reached values of ≈ 7.5 g.L^-1^ (R1) and ≈ 12 g.L^-1^ (R2), respectively, for each biological replicate, thus also presenting significant variability. Finally, among the other process parameters, Crl1 titres exhibited the highest variability between replicates, achieving values of 335 kAU·L^-1^ (R1) and 244 kAU·L^-1^ (R2).

As indicated in previous works (Garcia-Ortega et al., 2017; Gasset et al., 2022; Sales-Vallverdú et al., 2024), the hypoxic level had a significant impact on yeast physiology, causing relevant changes in central carbon metabolism. Therefore, significant differences in *RQ* due to a non-automated and non-accurate controller led to significant differences in biomass, Crl1 titre, ethanol production, and, ultimately, different process efficiencies and low reproducibility. These variations in *RQ* can be appreciated in Figure 3B. *RQ* was kept within the desired range (1.2 < *RQ* < 1.6), however, large oscillations and numerous peaks corresponding to each manual action on the stirring rate could be observed during the process. Additionally, during the first hours of both biological duplicates, *RQ* was not properly controlled at the desired set-point. The reason is that during this period, both CO_2_ production and O_2_ consumption were very low, so small errors in gas analysis led to high errors in *RQ* calculation. On the other hand, in the second replicate (R2) the calibration of the gas analyser was verified after finishing the fermentation. For this reason, the *RQ* values shown in the graph and recalculated *a posteriori* are above the range defined as the set-point. This highlighted a possible point for improvement: the calibration of the gas analyser before the start of fermentation, which is necessary for proper *RQ* calculation.

Interestingly, in some cases, control actions were performed when deemed necessary rather than every hour; i.e., when *RQ* was > 1.6, even if agitation had been increased less than 1 hour earlier. Moreover, during the most operationally demanding time, agitation was increased stepwise by smaller but more frequent steps, aiming to obtain *RQ* values within a narrow band around the desired set-point. An example of this is the period from t = 10 h to t = 15 h in R1. During this time, there seemed to be fewer oscillations in *RQ*. This could indicate what was evident from the outset, namely, that smoother and more frequent control actions lead to improved controller performance. This indicates a potential advance in the development of a more effective *RQ* control strategy if automated strategies could be implemented.

### 3.3 Automation of *RQ* control: integration of external signals and development of a Boolean Logic Controller (BLC)

Having identified the main shortcomings of the manual control strategy, the next step was the implementation of an automated control strategy. In essence, the idea was to automate the same control strategy. Ideally, the overall control system strategy had to be capable of calculating the *RQ* with high precision before deciding whether to increase agitation. This decision had to be fully automated, allowing the performance of smoother and more frequent control actions, resulting in a finer *RQ* control, thus avoiding major fluctuations (Brignoli et al., 2020; Craven et al., 2014).

Therefore, a software platform was required that could integrate data from both the bioreactor and gas analysers, process this data to accurately calculate the *RQ*, and subsequently execute control actions based on a simple rule (i.e., if *RQ* > *RQ* set-point, increase agitation).

Considering this, the implementation of so-called soft sensors is crucial (Mandenius and Gustavsson, 2015). Simple/basic soft sensors considering *CER*, *OUR,* and *RQ* based on first-principle models are among the most widely used in bioprocessing, being soft sensors to determine *μ* and *q*_*S*_ are frequently described (Barrigón et al., 2012; Luttmann et al., 2012; Stanke and Hitzmann, 2013; Rathore et al., 2021). Moreover, much literature is available on the use of soft sensor measurements to implement control strategies (Allampalli et al., 2022; Beiroti et al., 2019; Brunner et al., 2021; Randek and Mandenius, 2018; Sagmeister et al., 2013).

*Eve* software from Infors (Bottmingen, Switzerland) was selected for this purpose as it allows an easy implementation of soft sensors. Thus, data coming from the bioreactor and also from external devices (gas analyser, substrate addition pump, and ethanol sensor) were integrated into *Eve*, which acted as a “Supervisory Control And Data Acquisition” or *SCADA* system (Brunner et al., 2021; Ivarsson, 2017). Therefore, all data was available for the soft sensors and controller implementation in the *Eve* interface (Macdonald, 2018). In this current work, the calibration values of the gas analysers and the humidity measurements could be incorporated into the *RQ* calculation to improve its accuracy, upon which bioprocess control was based. Table 1 includes a list of all primary or direct variables and the derived or calculated variables, together with the *RQ* soft sensor implemented.

**TABLE 1:** Set of variables coming from the bioreactor and peripheral devices (primary variables) and calculations based on these measured variables (derived variables), available in the *Eve* interface, and soft sensor implemented using these variables as input data.

Primary variable	Origin	Description	Primary variable	Origin	Description
*TEMP*	Biostat B	Broth temperature	*Microburette Flowrate*	Microburette	Substrate flowrate
*JTEMP*	Biostat B	Jacket temperature	*EtOH Value* ^a^	Volatile (MetOH) sensor	Ethanol signal value
*STIRR*	Biostat B	Agitation rate	*CO*_*2*_	Gas analyser	%CO_2_ (internal calibration)
*pH*	Biostat B	pH	*O*_*2*_	Gas analyser	%O_2_ (internal calibration)
*pO*_*2*_	Biostat B	Dissolved oxygen (DO)	*Humidity*	Gas analyser	% Humidity
*BASET*	Biostat B	NH_4_OH 15% Added	*EXT A*	Gas analyser	CO_2_ sensor signal
*GF_AIR*	Biostat B	Air flowrate	*EXT B*	Gas analyser	O_2_ sensor signal
*GF_O2*	Biostat B	Pure oxygen flowrate			

Derived variable	Inputs	Outputs	Description
*ExitCO*_*2*_	*EXT A*	*Off-gas %CO*_*2*_	Concentration of CO_2_ in the off-gas. Sensor signal multiplied by external calibration values
*ExitO*_*2*_	*EXT B*	*Off-gas %O*_*2*_	Concentration of O_2_ in the off-gas. Sensor signal multiplied by external calibration values
*Volume*	*Microburette flowrate*	*Volume*	Volume estimation considering substrate addition (for OUR and CER calculation)
*Ethanol*	EtOH Value*	*Ethanol concentration*	Ethanol concentration calculation using MetOH sensor with mean calibration values

Soft sensor	Inputs	Outputs	Description
*RQ*	*Exit* *CO*_*2*_ *, Exit O*_*2*_ *, Humidity, GF_AIR, GF_O2, Volume*	*CER, OUR, RQ*	CER, OUR, and RQ calculation

^a^ Although a methanol sensor was used for ethanol measurement, the process variable was named “EtOH Value”.

The control strategy was built using the same principles as the manual control strategy, with three key upgrades: 1) an increase in control action frequency, 2) a reduction in ∆rpm, and 3) the ability to reduce the agitation rate if needed. However, in this case, the control algorithm was designed to be as automated as possible. Thus, the same control law as for the manual control (If *RQ* > *RQ* set-point, increase the agitation) was used. In this case, however, the control law was defined as a Boolean-logic controller (BLC), having only TRUE or FALSE values. The term “Boolean-logic control” has been used with similar control approaches (Premier et al., 2011). When *RQ* > 1.4, the control law was TRUE and thus agitation was increased.

The first improvement was the frequency of control actions, which was increased from every hour to every 10 min. This frequency was defined based on the response time of the system, from the moment the action on agitation was performed until the subsequent change was observed in off-gas composition, thus avoiding an eventual overacting performance of the controller (Simutis and Lübbert, 2015). The response time was estimated empirically, ranging between 5 and 10 min based on the results of manual control fermentations.

In line with the increase in frequency, the second improvement of the automated controller was the increase in rpm. This was drastically reduced to avoid big changes in *OTR* thus avoiding the high peaks and fluctuations in *RQ* observed with the manual control in Figure 3B. Additionally, to meet bioprocess requirements, the increase in agitation (∆rpm) was variable over time and changed from ∆rpm = 10, at the beginning of the feeding phase where the inertia of the system is slower, to ∆rpm = 25 at the end of the fermentation, where the dynamics were significantly faster.

The last improvement of the automated control strategy was the addition of a new functionality allowing for a decrease in agitation if the *RQ* was too low. For an *RQ* < 1.3, the controller applied an agitation rate modification of ∆rpm = −10. This upgrade contributed to the controller achieving greater precision and accuracy in the initial stages of the fed-batch because the arbitrarily selected initial agitation rate of 550 rpm would not be appropriate. However, apart from these initial hours, it should not be considered necessary since, as previously indicated, the inertia of the system is that *RQ* continuously increases as long as substrate addition, and thus the amount of total biomass, continues to increase.

**FIGURE 4:**
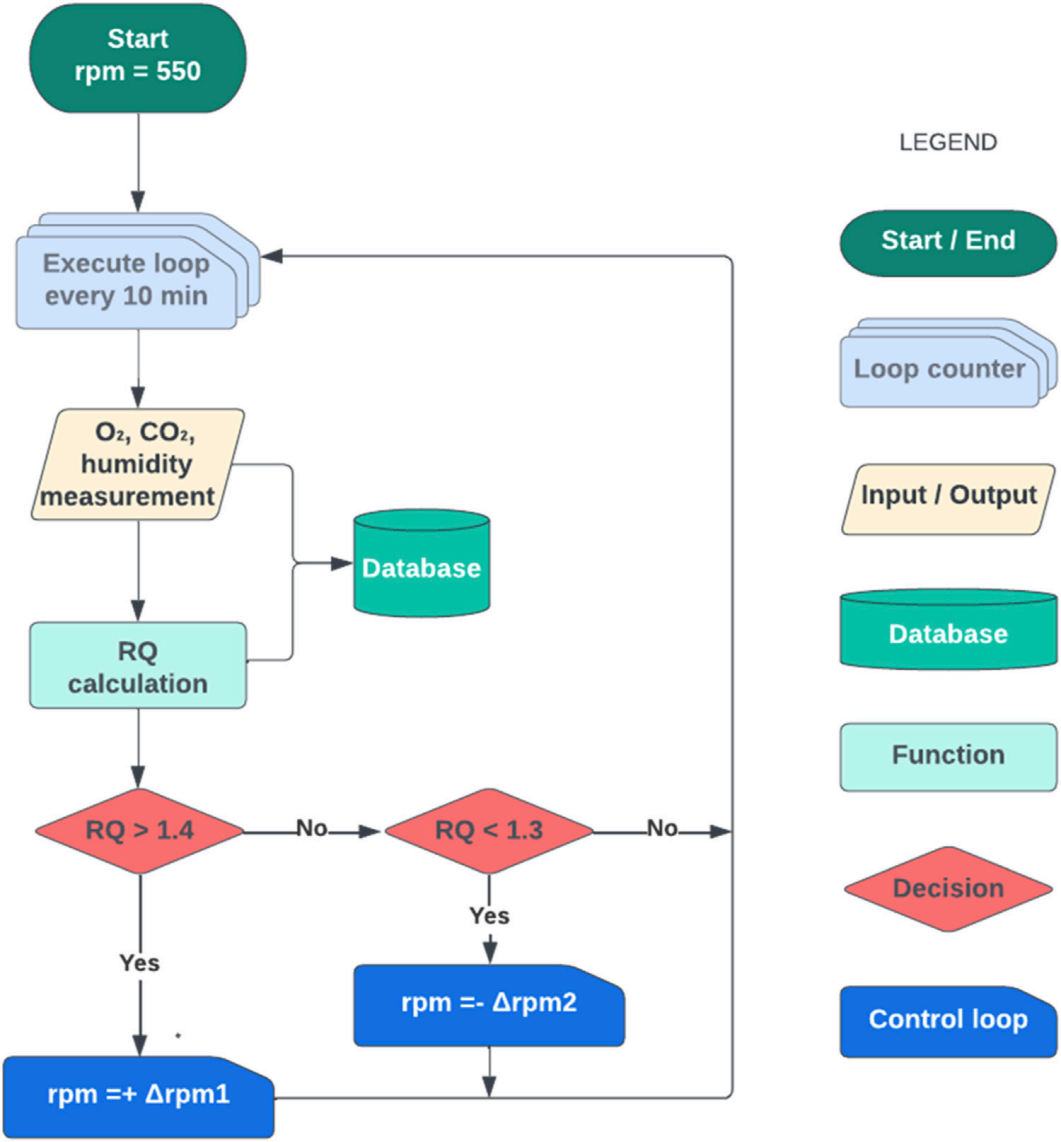
Flowchart of the Boolean-logic control (BLC) strategy. The initial agitation rate was set at 550 rpm, and every 10 min *RQ* was calculated by the *RQ* soft sensor, using O_2_ and CO_2_ measurements (including the calibration data of the analysers and the humidity in the inlet and off-gas streams). Then, if *RQ* > 1.4, the agitation rate was increased by 10/15/25 rpm (variable Δrpm1) and if *RQ* < 1.3, it was decreased by 10 rpm (Δrpm2). This control strategy was automated using *Eve* software, being necessary to only manually modify Δrpm1.

A flowchart of the Boolean-logic control algorithm can be seen in Figure 4.

Once the controller had been tuned, two biological replicates of a hypoxic fermentation were performed to test the efficiency of the controller. The fermentation strategy was the same as in the previous section (*µ* = 0.10 h^-1^), with the obvious exception of the *RQ* control strategy upgrade.

**FIGURE 5:**
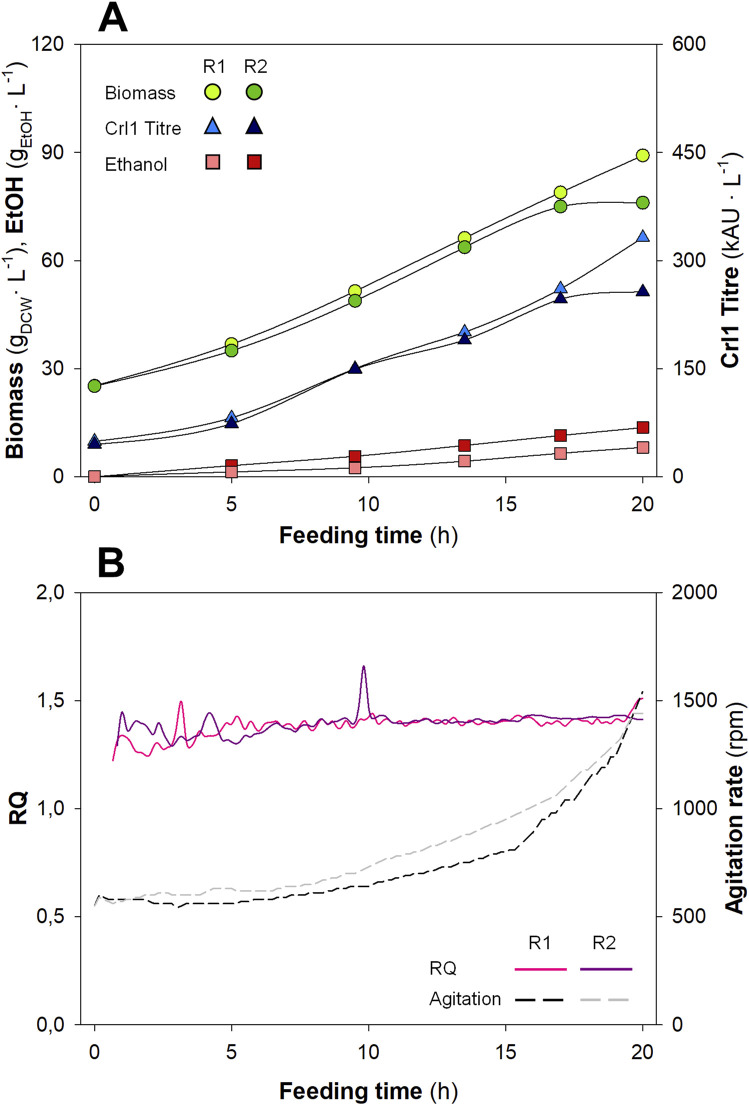
Key process parameters (Biomass, Ethanol, and Crl1), agitation rate, and *RQ* for the biological replicates (R1 and R2) with the Boolean-logic control strategy (BLC). **(A)** Biomass concentration (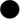, g_DCW_·L^-1^); Crl1 Titre (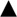, kAU·L^-1^); EtOH, Ethanol concentration (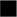, g·L^-1^). **(B)** Off-line *RQ* calculation (continuous lines); agitation rate (discontinuous lines, rpm).

The results of these fermentations are plotted in Figure 5. Practically identical biomass concentration, Crl1 titre, and ethanol concentration profiles can be observed in Figure 5A. However, in the last hours of the second replicate R2, biomass growth and Crl1 production were reduced, giving different biomass and Crl1 production for both replicates at the end of the feeding phase. Specifically, a final biomass and Crl1 titre of 89 g.L^-1^ and 332 KAU·mL^-1^, respectively, were achieved in R1, whereas the values were 76 g.L^-1^ and 257 KAU·mL^-1^ in R2. In the last 2 hours of R2, the specific growth rate could not be maintained at its set-point value, so *μ* < 0.10 h^-1^. Glucose accumulation up to 7.5 g.L^-1^ and higher ethanol concentrations were also observed in this second replicate. This could indicate that at high biomass concentrations and substrate addition, the process may become unstable.

Regarding controller efficiency, as can be observed in Figure 5B, an important improvement was found in terms of *RQ* control performance compared with the manual control results described in the previous section. In both replicates, the *RQ* was kept constant, within a narrow band around 1.4, with only small deviations of approximately ± 0.05. The only exception occurred within the first 1–2 h when the optimal agitation rate had to be found by the controller, having an initial input of 550 rpm. However, since the controller could increase and reduce the agitation rate, this optimal agitation was rapidly found.

The results of these experiments demonstrate that the process is highly reproducible in terms of *RQ* control. Moreover, discarding the last hours of R2, results also indicate that Crl1 production is more reproducible when implementing the automated rather than the manual controller, and suggest that the lack of *RQ* oscillations provides a better environment for cell growth and RPP (Xia et al., 2015). In fact, *RQ* oscillations could generate instability in terms of glucose metabolisation since fluxes through the oxidative and fermentative pathways are highly dependent on the level of oxygen limitation (Baumann et al., 2010). *RQ* oscillations can thus be considered as harmful as fluctuations in substrate concentration, which have been reported to affect production yields with *P. pastoris* as well as with other microbial cell factories (Junne et al., 2011; Lorantfy et al., 2013; Neubauer et al., 1995; Wang et al., 2020).

Thus, a very efficient *RQ* controller has been satisfactorily implemented, leading to a far more reproducible process that is slightly more efficient in terms of production and substrate utilisation. Based on the same heuristic rules as the manual control strategy, automation led to a more refined and more accurate *RQ* control and a much less labour-intensive production bioprocess. Nonetheless, there is still some room for improvement, namely developing a strategy with a variable ∆rpm (scheduled or even adaptive), so that it can be adjusted according to process requirements. The last control strategy implemented, presented in the next section, focuses on this point.

### 3.4 Implementing an Adaptive-Proportional Control (APC) using Artificial Intelligence (AI) algorithms: a foundation for industry 4.0

As the main goal and final step in designing and implementing the most efficient and reliable control strategy, aiming to achieve accurate and robust results, an innovative *RQ* controller based on AI algorithms was developed and successfully tested. The AI model for agitation rate prediction was trained using data obtained from all the hypoxic fermentation experiments previously described. In addition, fermentations conducted under hypoxic conditions at different *µ* were also used for the training process (Sales-Vallverdú et al., 2024).

As stated in the previous section, having a variable and adaptive ∆rpm was the next and final step in the development of an optimal control strategy to maintain the desired hypoxic conditions throughout the fed-batch hypoxic phase. It was previously determined that this parameter should increase throughout the process in accordance with exponential biomass growth. Strictly speaking, for a fully automated control strategy, the controller should be able to define the correct ∆rpm at any time during the process. Thus, for its relative simplicity and adjustability, an adaptive proportional (Adaptive-P) control was implemented.

As in the previous cases, the controlled variable was *RQ,* with a set-point of 1.4. It was calculated in the *Eve* environment using the same “*RQ*” soft sensor implemented for the BLC, such that the calibration values of the gas analysers could also be incorporated into the *RQ* determination. However, in this case the control of *RQ* was applied using the DT system.

The DT system for controlling the bioreactor consisted of several interconnected components that worked together to optimise the bioreactor process. At the core of the system was the cloud platform, known as the Aizon platform, which served as the central hub for data management, processing, and AI-driven analysis. The Aizon platform received data from the physical sensors and from the *Eve* soft sensor of the bioprocess via the on-premise element, called the BeDataFeeder. Communication with *Eve* software was facilitated through API REST calls, ensuring seamless integration and data exchange between the physical and digital components of the system. Once the data reached the Aizon platform, it underwent comprehensive processing and analysis by various AI models specifically designed for bioprocess control. Additionally, the platform hosted AI models for anomaly detection, which continuously monitored the bioreactor data for any deviations from normal operation. To enable communication between the BeDataFeeder and the Aizon platform, the MQTT protocol was utilised. This lightweight messaging protocol ensured efficient and reliable data transmission between the on-premise component and the cloud platform, facilitating real-time updates and control actions. Based on the inputs provided by the sensors, the adaptive control AI models on the Aizon platform dynamically adjusted the agitation rate to maintain the process within the desired parameters. These adaptive control algorithms leveraged machine learning techniques to continuously improve their accuracy and effectiveness over time.

**FIGURE 6:**
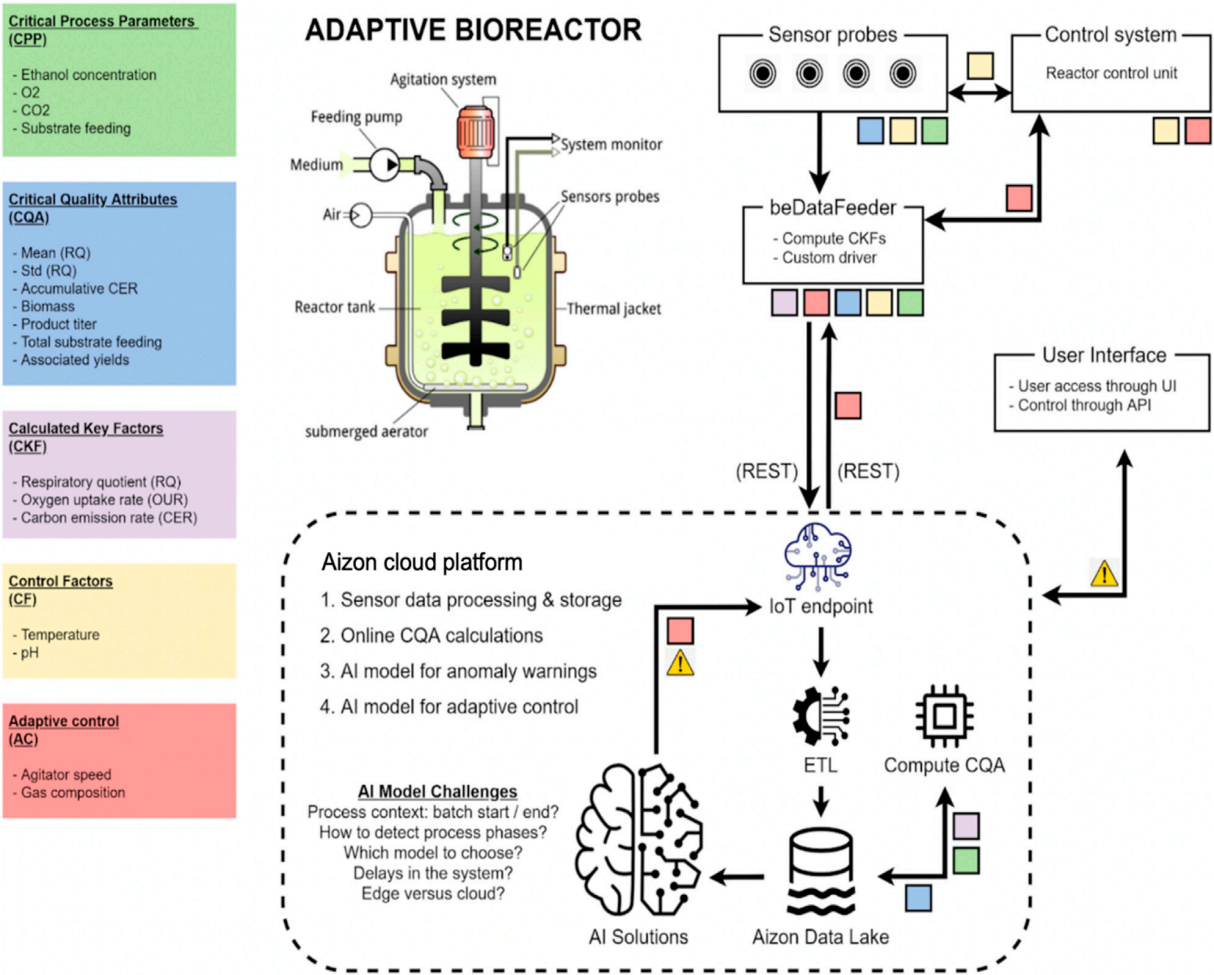
Global scheme of the AI-managed adaptiveness implementation managed in the hypoxic bioprocess of the *Pichia pastoris* cell factory.

Overall, the DT system combined on-premise data collection with cloud-based AI processing on the Aizon platform to provide real-time monitoring, predictive analysis, and adaptive control of the bioreactor process. By integrating advanced technologies and leveraging data-driven insights, the system aimed to optimise bioreactor operations and achieve consistent, high-quality batch outcomes. Moreover, the BeDataFeeder was equipped with a copy of the AI model and could store the data and upload it all at once if the connection to the cloud ever faltered. A scheme of the overall system is presented in Figure 6.

Overall, the controller applied Equation 6 to calculate ∆rpm, multiplying an adaptive P gain “K _p_” by the error between the *RQ* and the *RQ* set-point or “ε”, and sent this information to *Eve* to modify the agitation rate. All these steps were implemented in Python, using REST API calls to communicate with the *Eve* system.

$$∆rpm=\left(RQ-{RQ}_{set-point}\right)\cdot Adaptive\;P\;Gain=\varepsilon \cdot \;K_{P}$$ (6)

Interestingly, Equation 6 enabled the controller to lower the agitation if *RQ* < *RQ*_set-point_, ∆rpm becomes negative.

A new agitation rate was predicted every minute, however, its modification was only carried out with the minimum time interval between subsequent control actions of 7 min to account for the system’s time response, as detailed in the previous section.

**FIGURE 7:**
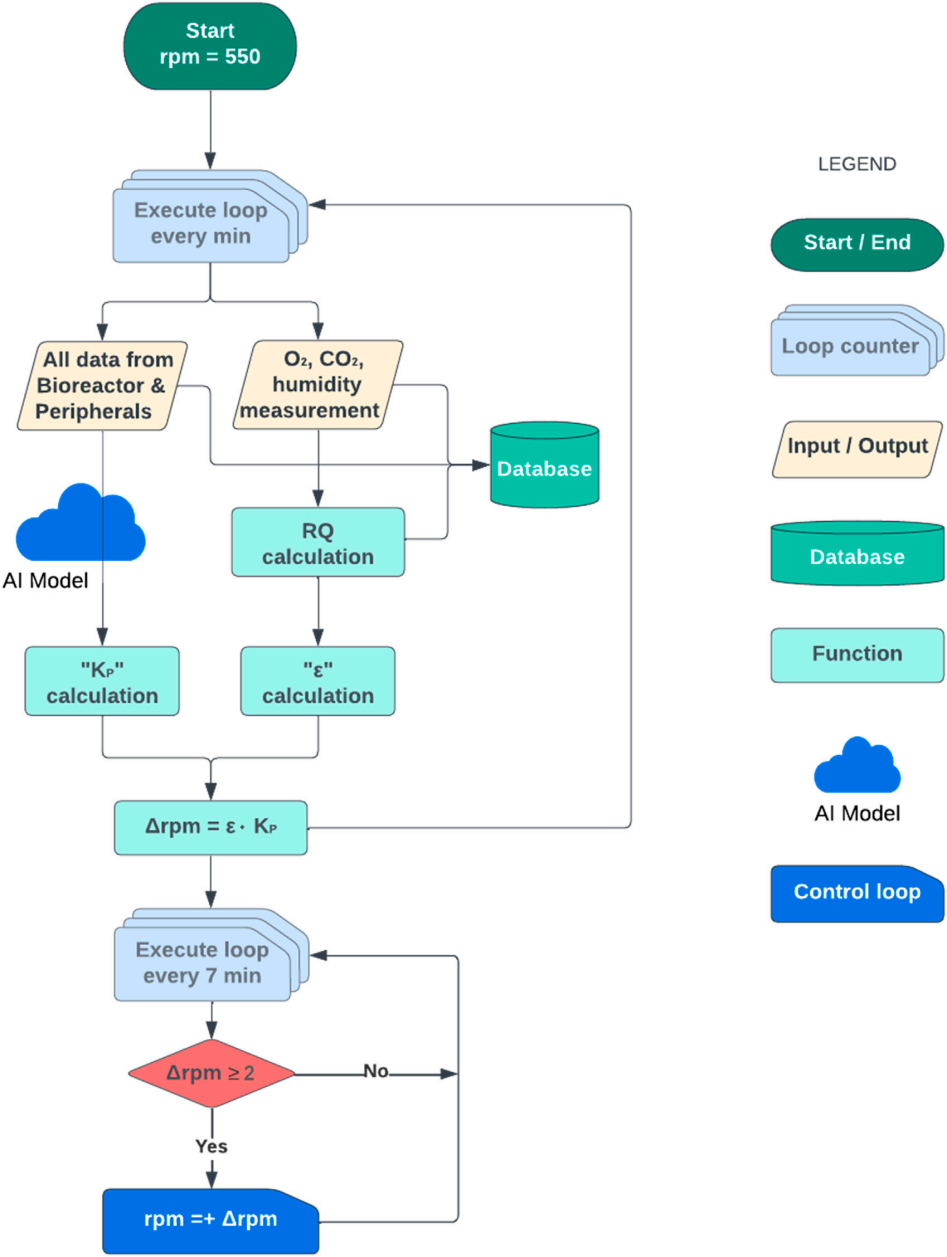
Flowchart of the AI-aided Adaptive Proportional Control (AI-APC) strategy. The initial agitation rate was set at 550 rpm, and *RQ* was calculated every min. Next, ε was calculated in parallel to K_P_, which was calculated through an AI model (random forest regression algorithm) using all data coming from the bioreactor and peripherals. Finally, Δrpm (positive or negative) was calculated using ε and K_P_ and agitation was modified every 7 min only if Δrpm ≥ 2.

Also, to avoid small but constant oscillations of the agitation rate, if the difference between the current and predicted agitation was less than 2 rpm (∆rpm < 2), this modification was not implemented, and the controller repeated this procedure in the following minute. Therefore, the frequency of control actions was variable, with intervals from at least 7 min to intervals of approximately half an hour, depending on the requirements of the process. The result was that both adaptive ∆rpm and frequency in control actions could be applied, thereby overcoming the main weaknesses of the BLC strategy. A simplified flowchart of the AI-aided Adaptive-P Control (AI-APC) algorithm is shown in Figure 7.

**FIGURE 8:**
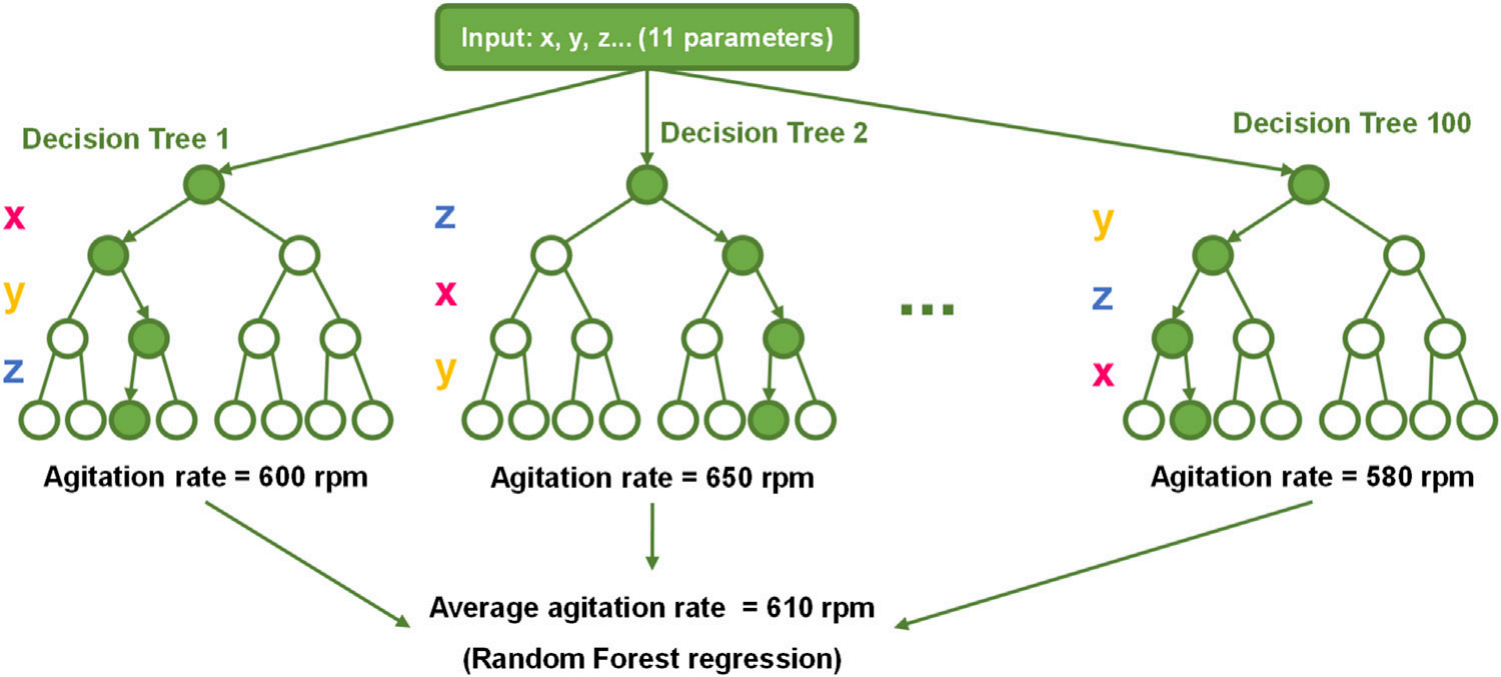
Scheme of a random forest regressor algorithm. One hundred decision trees, each one trained with a different subset of data, predict the necessary agitation rate to maintain *RQ =* 1.4, and the final value is obtained by averaging all predictions. In addition, each decision tree may assign different priorities to each parameter or process variable, depending on the subset of data used in its training process. x, y, and z represent different variables or process parameters.

The calculation of the “Adaptive-P Gain” parameter or “K_P_” was achieved through the application of AI algorithms. Specifically, a random forest algorithm was applied. The random forest algorithm is a machine-learning technique that seeks to make accurate predictions by leveraging the power of multiple decision trees. During training, each decision tree divides the dataset and saves the rule used to make the division as a node. When a subset of data is compact enough depending on the task at hand (that all samples belong to the same class for classification and that all samples have the target variable within a relatively small range for regression tasks), a leaf node is created, and the prediction related to this node is saved. To avoid having the exact same tree N times, the training algorithm passes only a subset of features to each tree, forcing them to choose different division rules. The mechanism of operation of the algorithm is schematically represented in Figure 8.

The random forest algorithm was selected due to its robustness when presented with scenarios not present in the training, thanks to the ensemble nature of the model. Also, because each decision tree is slightly different, it is harder to overfit the data. Furthermore, it is excellent for modelling nonlinear correlations such as those encountered in bioprocesses, and it has already been used in bioprocess engineering applications (Mowbray et al., 2021; Singh and Singhal, 2022).

In this case, 100 decision trees were implemented. Thus, when making a prediction, the algorithm passed the input through each decision tree, and the results from all decision trees were combined to form a final prediction. Concretely, when the algorithm was trying to predict a numerical value, such as the optimal agitation rate, the average of the predicted values from all decision trees was taken (Breiman, 2001).

Additionally, to further evaluate the performance of the AI-APC controller towards a disturbance in the system, the inlet gas composition was modified at t = 13.3 h of the feeding phase in both replicates. At that point, the airflow rate was automatically modified from 2.0 L.min^-1^ to 1.8 L.min^-1^, and a flow rate of 0.2 L.min^-1^ of pure oxygen was added, giving an inlet gas oxygen concentration of 28.87%. A reduction in the agitation rate was therefore expected at this point, although the nature of the response was still unknown.

Once the controller had been implemented and tested with simulated hypoxic conditions, two hypoxic fermentations were conducted to check whether the AI-based controller could provide better results than the BLC. As in the previous control strategy, both fed-batches were conducted with a pre-programmed exponential feeding profile of glucose with *μ* = 0.10 h^-1^ and an *RQ* set-point = 1.4.

**FIGURE 9:**
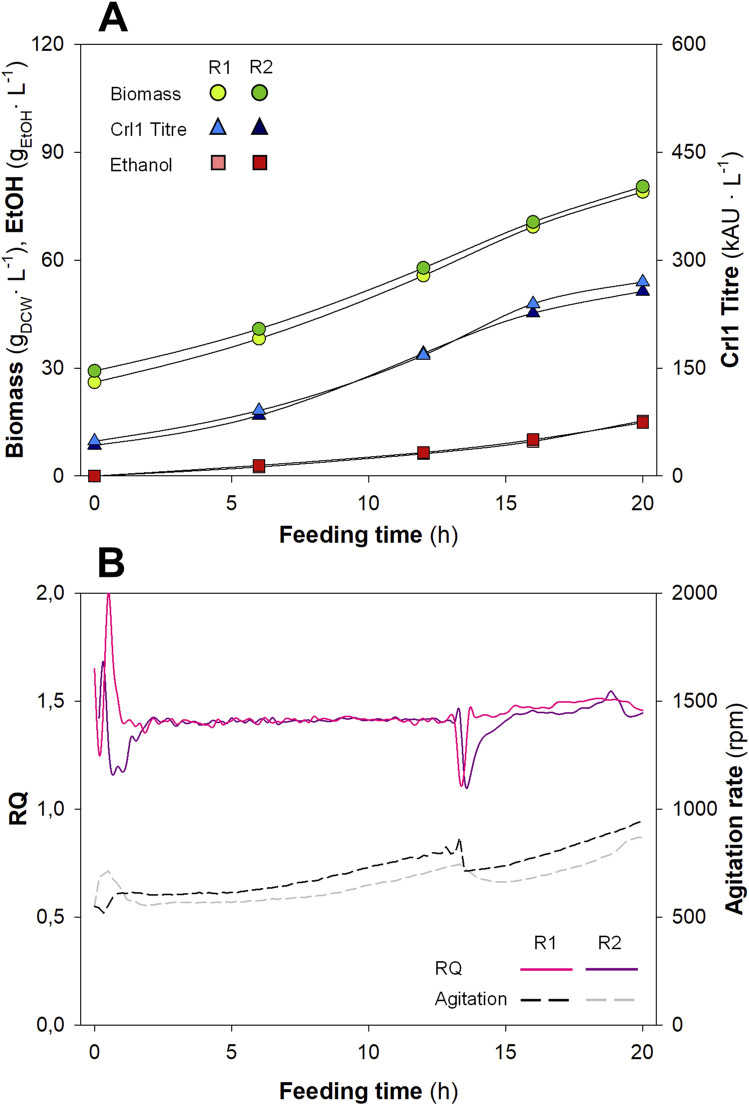
Key process parameters (Biomass, Ethanol, and Crl1), agitation rate, and *RQ* for the biological replicates (R1 and R2) with the AI-aided Adaptive Proportional Control (AI-APC) strategy. **(A)** Biomass concentration (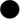, g_DCW_·L^-1^); Crl1 Titre (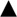, kAU·L^-1^); EtOH, Ethanol concentration (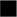, g·L^-1^). **(B)** Off-line *RQ* calculation (continuous lines); agitation rate (discontinuous lines, rpm).

Figure 9 illustrates the results of these two AI-controlled fermentations. Regarding biomass concentration, the Crl1 titre and ethanol production of both replicates, shown in Figure 9A, were very similar to those obtained with the BLC. They were particularly comparable between them, reaching values of 79 g_DCW_·L^-1^ (R1) and 80 g_DCW_·L^-1^ (R2) of biomass, 257 kAU·L^-1^ (R1) and 270 kAU·L^-1^ (R2) of Crl1 titre, and 15 g.L^-1^ (R1 and R2) of ethanol at the end of fermentation. This highlights the good reproducibility achieved with this control strategy. However, in both replicates the biomass production decreased slightly in the last hours compared with the previous set of fermentations with the BLC. This could be explained by a small increase in *RQ* in the last hours, which led to a reduction in *Y*_*X/S*_ and, thereby, a lower biomass generation as higher ethanol profiles were also observed.

In Figure 9B, which plots the agitation and *RQ* profiles, a very precise *RQ* control can be observed, which is very close to the set-point and exhibits very small oscillations. This strategy performed at least as well as the BLC in terms of controller efficiency, but it required significantly less time commitment than with the BLC and demanded far less effort than with the MHC. Since ∆rpm was automatically predicted every minute and agitation was automatically modified every seven (or more) minutes, it was not necessary to assess whether the ∆rpm was appropriate at each point in the process, and the researcher only had to be in the laboratory for sampling and supervising.

It is noteworthy that all data sets used to train the model included the results from the fermentations performed with the MHC and BLC, included in this work, and some previously mentioned fermentations performed with different *μ* and detailed in the bibliography, where pure oxygen was not used (Sales-Vallverdú et al., 2024). Thus, it makes sense that during the last third of the cultivations controlled with the AI-APC, when inlet air was enriched with pure oxygen, the controller did not regulate the *RQ* as efficiently as during the rest of the feeding phase. Even so, the *RQ* deviation was very slight, only about 0.1 *RQ* units above the set-point. On the other hand, the controller’s response towards a disturbance was very fast, being able to return the *RQ* to the set-point in less than 1 h for R2 and less than 30 min for R1.

As commented in the previous section, the high *RQ* stability led to a more reproducible bioprocess performance, including Crl1 production as well as lower byproduct generation, including ethanol, arabitol, and succinate, compared with the manual control strategy.

### 3.5 Performance comparison between the control strategies implemented

To evaluate the correct performance of each *RQ* controller and the improvement when applying novel AI-based strategies, a comparison of the three control strategies implemented in terms of bioprocess performance and reproducibility was conducted.

Table 2 shows the numerical values of key process parameters obtained in each hypoxic fermentation. Additionally, the mean value and standard deviation (SD) are shown. Similar values for key process parameters were observed for all strategies, although an undesired reduction in biomass production and thus a reduction in *μ* was detected with the third strategy, as mentioned in the previous section. This could be explained by the increase in *RQ* observed in the last third of the feeding phase, which led to an inversely proportional decrease in *Y*_*X/S*_. As mentioned, ethanol production was also higher, reflected in the *q*_*EtOH*_ values in Table 2.

**TABLE 2:** Value of key process parameters obtained in fed-batch hypoxic fermentations with the three control strategies tested. Specific growth rate, *µ* (h^-1^); specific substrate consumption rate, *q*_*S*_ (g_S_·g_DCW_^-1^·h^-1^); biomass-to-substrate yield, *Y*_*X/S*_ (g_DCW_·g_S_^-1^); specific ethanol production rate, *q*_*EtOH*_ (g_EtOH_·g_DCW_^-1^·h^-1^); respiratory quotient, *RQ*; specific Crl1 production rate, *q*_*P*_ (AU·g_DCW_^-1^·h^-1^); and product-to-biomass yield, *Y*_*P/X*_ (kAU·g_DCW_^-1^). Values in bold represent the mean and SD (±) between biological replicates (R1 and R2).

	MHC		BLC		AI-APC	
	R1	R2	R1	R2	R1	R2
*μ* (h^-1^)	0.105	0.096	0.103	0.097	0.095	0.091
	**0.101 ± 0.006**		**0.100 ± 0.004**		**0.093 ± 0.003**	
*q*_*S*_ (g_S_·g_DCW_^-1^·h^-1^)	0.21	0.22	0.22	0.21	0.22	0.21
	**0.21 ± 0.01**		**0.22 ± 0.01**		**0.22 ± 0.01**	
*Y*_*X/S*_ (g_DCW_·g_S_^-1^)	0.50	0.45	0.47	0.45	0.43	0.43
	**0.47 ± 0.04**		**0.46 ± 0.01**		**0.43 ± 0.01**	
*q*_*EtOH*_ (g_EtOH_·g_DCW_^-1^·h^-1^)	0.020	0.034	0.027	0.030	0.040	0.037
	**0.027 ± 0.010**		**0.029 ± 0.002**		**0.038 ± 0.002**	
*RQ*	1.41	1.53	1.38	1.38	1.40	1.44
	**1.47 ± 0.09**		**1.38 ± 0.01**		**1.42 ± 0.02**	
*q*_*P*_ (AU·g_DCW_^-1^·h^-1^)	348	310	335	358	317	311
	**329 ± 27**		**347 ± 16**		**314 ± 4**	
*Y*_*P/X*_	3.60	3.42	3.83	3.92	3.67	3.83
(kAU·g_DCW_^-1^)	**3.51 ± 0.12**		**3.88 ± 0.06**		**3.75 ± 0.12**	

It is worth noting that *RQ* values from Table 2 are the mean *RQ* values throughout each fermentation. As a result, the mean and SD values shown in bold are indicative of the variability between replicates.

Thereby, in terms of bioprocess reproducibility, the application of two more sophisticated *RQ* controllers led to a reduction in variability between replicates, particularly concerning Crl1 production, since the SD was smaller for BLC and AI-APC.

However, to assess the controller’s efficiency, its autonomy, accuracy, and precision should also be considered. Regarding autonomy, the manual control strategy was clearly the least efficient. When comparing BLC and AI-APC, in the former, several ∆rpm updates were required during cultivation, whereas in the latter, this change was applied automatically in accordance with the prediction of an AI model, therefore making it the most autonomous.

In addition, the *RQ* deviation from its set-point highlights the controller’s accuracy, whereas the deviation from the mean *RQ* can be considered an estimator of the controller’s precision. With this aim, two independent statistical performance indicators were calculated to assess this accuracy and precision: Mean Relative Error (MRE) and Root Mean Square Deviation (RMSD), which are defined by Equations 7, 8. MRE can be considered as a relative value of a mean *RQ* error through the fed-batch phases, having values between 0 (good performance) and 1 (bad performance), and it was used to assess the accuracy of the controllers. On the other hand, RMSD could be seen as a mean SD between *RQ* values throughout the fed-batch phases and the mean *RQ* value of each fermentation, shown in Table 2, and it was used to determine the precision of the controller.

$$MRE=\frac{1}{n}\sum_{i=1}^{n}\frac{\left|y_{i}{-y}_{sp}\right|}{y_{sp}}$$ (7)

$$RMSD=\sqrt{\sum_{i=1}^{n}\frac{{\left(y_{i}-ȳ\right)}^{2}}{n}}$$ (8)

Where *y*_*i*_ is the value of the variable (*RQ*) at each time-point (_*i*_), *y*_*sp*_ is the *RQ* set-point, since the scope is to evaluate the accuracy of the controller (MRE), and *ȳ* is the mean *RQ*, for the analysis of the controller’s precision (RMSD). They can afford redundant information, however, the analysis of two different statistics can help avoid data artefacts.

The values obtained using all data points from each pair of biological replicates are shown in Table 3.

**TABLE 3:** Value of Mean Relative Error (MRE) and Root Mean Square Deviation (RMSD) for each hypoxic fed-batch fermentation with the three control strategies tested.

		MHC		BLC		AI-APC	
		R1	R2	R1	R2	R1	R2
MRE	Accuracy (how close is *RQ* to the set-point)	0.049	0.141	0.045	0.046	0.035	0.040
RMSD	Precision (how close are *RQ* measurements to each other)	0.083	0.194	0.108	0.112	0.083	0.105

Based on the analysis of these statistics, in terms of accuracy, the last two strategies outperformed the manual control strategy, having lower MRE values. These numbers indicate that the mean error was about 5% (R1) and 14% (R2) with the MHC, about 4.5% with the BLC, and <4% with the AI-APC strategy. On the other hand, in terms of precision, the same trend was observed when comparing RMSD values. As mentioned previously, RMSD could be considered a mean SD from the mean *RQ* value, becoming lower as the complexity of the controller increases.

In addition, as mentioned before, during the last third of the AI-APC fermentations, the *RQ* was constantly above the set-point, suggesting that *RQ* control during the rest of the fed-batch was more effective. This point added to the fact that in those fermentations there was a disturbance in the inlet gas composition, which had a direct impact on *RQ* and, therefore, also on MRE and RMSD, making the AI-APC strategy the most efficient.

In summary, the application of AI in CPV for monitoring and controlling the fermentation process of *Pichia pastoris* is invaluable. By leveraging AI, we can detect anomalies in real-time and ensure that every batch meets the highest quality standards under the established conditions (Ondracka et al., 2023), ultimately enhancing the reliability and efficiency of the biomanufacturing process by integrating Critical Process Parameters (CPP) as input variables to the AI model, the optimal agitation speed of the stirrer in the bioreactor is continuously adjusted as the target variable, 24/7 during all batches, ensuring a high yield.

## 4 Conclusion

This work presents the implementation of advanced control strategies in microbial fermentation fed-batch cultures to enhance bioprocess performance, increasing the robustness and reproducibility of the cultivations. When implementing the innovative physiological control based on *RQ,* initially, a Boolean-Logic Controller (BLC) was implemented to address deviations observed with the Manual-Heuristic Control strategy (MHC), resulting in improved reproducibility. Subsequently, an innovative AI-aided Adaptive-Proportional Control strategy (AI-APC) was developed using a random forest algorithm, delivering highly satisfactory results. Both strategies significantly improved control accuracy, with AI-APC showing greater promise. The integration of measurement devices into a SCADA system, coupled with a cloud-stored DT of the fermenter, facilitated these advancements.

Overall, these two strategies mark notable progress in enhancing bioprocess control efficiency and reliability. However, in terms of adaptability and automation, the AI-APC clearly outperformed the previous ones since the performance towards a disturbance was extremely rapid and precise, proving AI to be a tool that can be efficiently used in bioprocess control, representing a successful application of AI in a field where such technologies are underutilised.

The content and successfully fulfilled goal of this article, not only showcase the pivotal role of DT systems such as Process Analytical Technologies (PAT) during the CPV phase of drug manufacturing but also serve as a compelling demonstration of the potent capabilities of AI at the helm of autonomous DT. By seamlessly integrating with manufacturing processes, these AI-driven DTs offer real-time insights and predictive analytics, ensuring precision, efficiency, and compliance in bioprocesses. This convergence of cutting-edge technologies underscores the transformative potential of AI-powered solutions in biotechnology manufacturing, pointing towards a new era of innovation and optimisation in the bioindustry.

## Data Availability

The raw data supporting the conclusions of this article will be made available by the authors, without undue reservation.
